# Combined Preoperative LMR and CA125 for Prognostic Assessment of Ovarian Cancer

**DOI:** 10.7150/jca.42477

**Published:** 2020-03-04

**Authors:** Ying Tang, Hui-quan Hu, Fang-xiang Tang, Dan Lin, Rui Shen, Li Deng, Ya-lan Tang, Li-hong Deng, Mi Zhou, Jun Li, Bin Su, Fan Xu

**Affiliations:** 1 Department of Obstetrics and Gynecology, The Affiliated Nanchong Central Hospital of North Sichuan Medical College, Nanchong, Sichuan, PR China; 2 North Sichuan Medical College, Nanchong, Sichuan, PR China; 3 Department of Computer Science and Technology, School of China West Normal University, Nanchong, Sichuan, PR China

**Keywords:** ovarian cancer, lymphocyte-to-monocyte ratio (LMR), CA125, prognosis

## Abstract

**Objectives**: To investigate the role of inflammation-related factors, lymphocyte-to-monocyte ratio (LMR) alone and combined detection with cancer antigen 125 (CA125), in the prognostic assessment of ovarian cancer (OC).

**Methods**: A retrospective clinicopathologic review was performed. The receiver-operating characteristic (ROC) curves of LMR, CA125, and COLC predicting mortality in OC patients were constructed. Besides, Kaplan-Meier and Cox logistic regression models were used to plot the survival curves and determine the independent prognostic factors.

**Results**: A total of 214 OC patients were identified in this cohort. The mean duration of follow-up was 64 months (minimum 8 months, maximum 116 months). In this cohort, 135 cases died (63.1%), and the median progression-free survival (PFS) and overall survival (OS) were 20 and 39.5 months, respectively. Results of the multivariate Cox regression model showed that LMR≤3.8 (HR = 0.494, 95% CI: 0.329-0.742, P = 0.001) and CA125>34 U/ml (HR = 1.641, 95% CI: 1.057-2.550, P = 0.027) were significantly associated with poor PFS; and LMR≤3.8 (HR = 0.459, 95% CI: 0.306-0.688, P = <0.001) and CA125>34 U/ml (HR = 1.946, 95% CI: 1.256-3.015, P = 0.003) were significantly associated with OS. Furthermore, the area under the curve of COLC was higher (0.713) than that of LMR (0.709) or CA125 (0.583), the specificity of COLC was higher (75.9%) than that of LMR (62%) or CA125 (40.5%) in predicting mortality in OC patients.

**Conclusions**: LMR alone and combined with CA125 might be used as predictive markers in OC. Furthermore, as a prognostic factor, COLC might have a higher specificity to predict the outcome.

## Introduction

Ovarian cancer (OC) is the deadliest gynecological cancer, and threats women's health significantly 1. Siegel RL 2 reported that 13,980 OC deaths are projected to occur in the United States in 2019. Although the OC death rate has declined annually 2-4, the prognosis of OC remains disappointing 5-6. Various studies have identified that biomarkers could have the greatest impact on survival results in prognostication 1, 7.

Cancer antigen 125 (CA125), considered as a tumor-related maker, is a glycoprotein tumor- associated antigen, which exists in the tissues and serum of epithelial ovarian cancer. It is one of the most studied factors in OC, and its role in predicting the prognosis of OC has been evaluated 8-9. However, it is not widely used in clinic due to its relatively low specificity 7.

As an inflammation-related prognostic factor, the lymphocyte-to-monocyte ratio (LMR) represents the lymphocyte levels divided by the monocyte levels in OC, and it has recently been evaluated for its ability to predict the survival of patients with various solid cancers 10-12. Researchers have found that LMR might be a prognostic factor of OC 13-14; however, consensus has not been reached due to inconsistent results of different studies 15.

Consequently, we attempted to evaluate the prognostic ability of LMR combined with CA125 (COLC) to investigate the role of LMR and COLC in predicting progression-free survival (PFS) and overall survival (OS) in OC patients.

## Materials and Methods

### Patients

A total of 214 patients with OC who underwent initial cytoreductive surgery between January 2006 and December 2016 in the affiliated Nanchong Central Hospital of North Sichuan Medical College were enrolled.

Inclusion criteria were as follows: histologically confirmed ovarian cancer; histologic subtype, histological grade, lymph node metastases of OC were obtained by histologic examination; and histologic reports were reviewed and diagnosed by two senior pathologists.

Exclusion criteria were as follows: severe liver and kidney damage, autoimmune diseases, thrombus and hemorrhagic diseases, second malignancies or multiple primary malignancies, infectious diseases in the past 2 weeks, chemotherapy treatment or other anti-tumor treatment and no blood routine examination results within 3 days before surgery.

The standard procedures for primary debulking surgery (PDS) consisted of total hysterectomy, adnexectomy, appendectomy, omentectomy, pelvic and para-aortic lymphadenectomy sampling, routine abdominal washing and any tumorectomy of metastatic lesions if applicable 4, 16. Postoperative adjuvant chemotherapy (ACT) consisted of paclitaxel (175 mg/m2), and carboplatin (AUC 5) was initiated within 6 weeks of PDS, repeated every three weeks for 6 cycles when required 16. Patients with advanced stage (FIGO Ⅲ∼Ⅳ), of diseases who have received platinum-based neoadjuvant chemotherapy (NACT) followed by interval debulking surgery (IDS) were also included in this study 16. Optimal debulking surgery (ODS) was defined as completion of cytoreduction resulting in residual tumor less than 1 cm in diameter 17.

The primary endpoint of the study was PFS, which was calculated from the date of surgery to the date of recurrence or progression 18. The secondary endpoint of the study was OS, which was defined as the time from the date of surgery to the date of death or last follow-up 18. All OC patients were followed up after the end of chemotherapy: every 3 months for the first 2 years, every 6 months for 2-5 years, and then annually for 5 years. At each visit, the patients were assessed by ultrasound examination of the pelvic cavity, abdomen (liver, gallbladder, pancreas, and spleen), serum CA125 level test, and CT or MRI examination of relevant sites when necessary.

This study was approved by the Ethics Committee of the affiliated Nanchong Central Hospital of North Sichuan Medical College. Because of the retrospective study design, informed consent could not be obtained from each patients. Instead of obtaining informed consent from each patient, we posted a notice about the study design and contact information at a public location in the affiliated Nanchong Central Hospital of North Sichuan Medical College.

### Methods

All of the following data were obtained from medical records: age, BMI, FIGO stage, tumor (histological grade and type), attainment of optimal debulking, whether combined with malignant ascites and lymph node metastases, clinical characteristics (CA125, lymphocyte, and monocyte levels) within 2 days before surgery. Based on previous studies, LMR was defined as the absolute lymphocyte count/the absolute monocyte count 18.

Statistical analyses were performed with SPSS software (ver. 20.0; IBM Corp, Armonk, NY, USA). The optimal cutoff values for LMR and CA125 were determined via the receiver-operating characteristic (ROC) curve, and the OC patients were categorized into two groups according to the optimal cut-off value of LMR and CA125, respectively. The definition of COLC was according to the Glasgow prognostic score (Table 4). The areas under curves (AUCs) were compared using ROC curves calculated for LMR, CA125 and COLC. The ROC was performed with MedCalc software (ver. 15.2.1; MedCalc Software bvba, Ostend, Belgium). The PFS and OS of the 2 groups were compared statistically. Univariate and multivariate Cox regression analyses were used to analyze the factors that might affect PFS and OS in OC patients. Differences in survival among classification groups were analyzed using Kaplan-Meier curves and log-rank tests. A two-sided P value < 0.05 was considered statistically significant.

## Results

### Patient characteristics

A total of 214 OC patients were identified. Their median age was 50 years (range: 20-88 years). The majority histologic subtype and grade of OC patients were serous and G3 (both n = 146, 68.2%), and there were 101 people (47.2%) were classified into Federation of Gynecologists and Obstetricians (FIGO) stage Ⅲ at initial diagnosis. The mean duration of follow-up was 64 months (minimum 8 months, maximum 116 months). In this cohort, 135 cases died (63.1%); and the median PFS and OS were 20 months and 39.5 months (table 1), respectively. The baseline characteristics of all OC patients are listed in table 1.

### ROC Curves of the LMR and CA125

**Figure 1:**
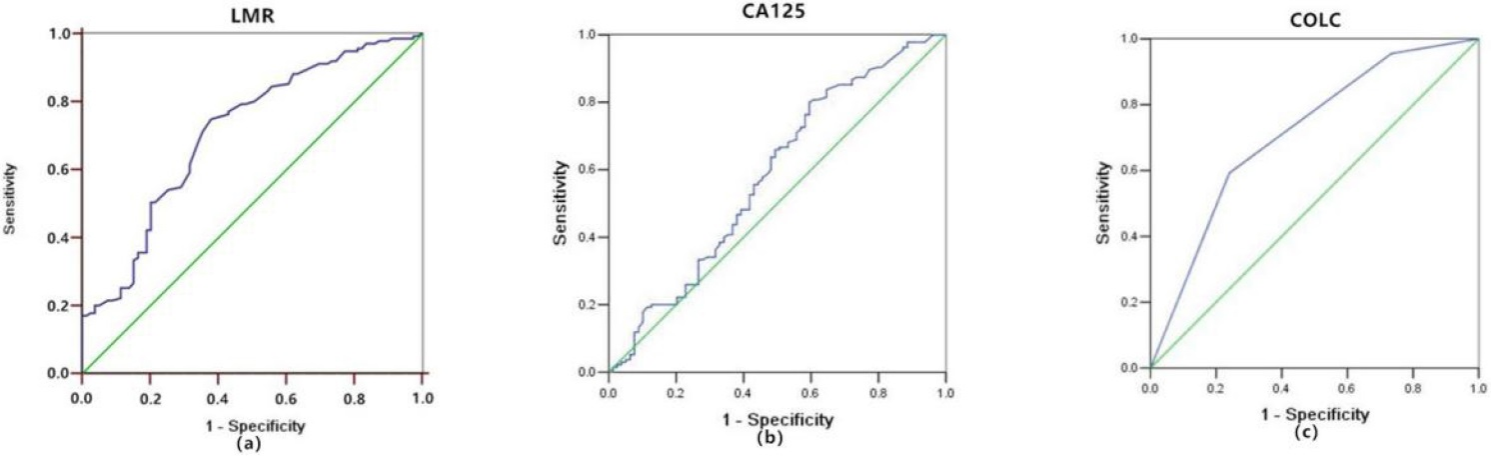
Receiver-operating characteristic curve analyses of LMR (a), CA125 (b), and COLC (c) in OC patients. LMR, lymphocyte/monocyte ratio; CA125, cancer antigen 125; COLC, combination of LMR and CA125.

The ROC curve of LMR predicting mortality in OC patients was shown in Figure 1a. Optimal cutoff point of LMR was 3.8, the sensitivity and specificity were 74.8% and 62%. OC patients were divided into two groups based on this cutoff value (LMR > 3.8, n = 83, 38.8%; LMR ≤ 3.8, n = 131, 61.2%).

The ROC curve of CA125 predicting mortality in OC patients was shown in Figure 1b. The optimal cutoff point of CA125 was 34 U/ml, the sensitivity and specificity were 80% and 40.5%. OC patients were divided into two groups based on this cutoff value (CA125 >34 U/ml, n =155, 72.4%; CA125 ≤34 U/ml, n = 59, 27.6%).

### Survival and prognostic factors

**Figure 2:**
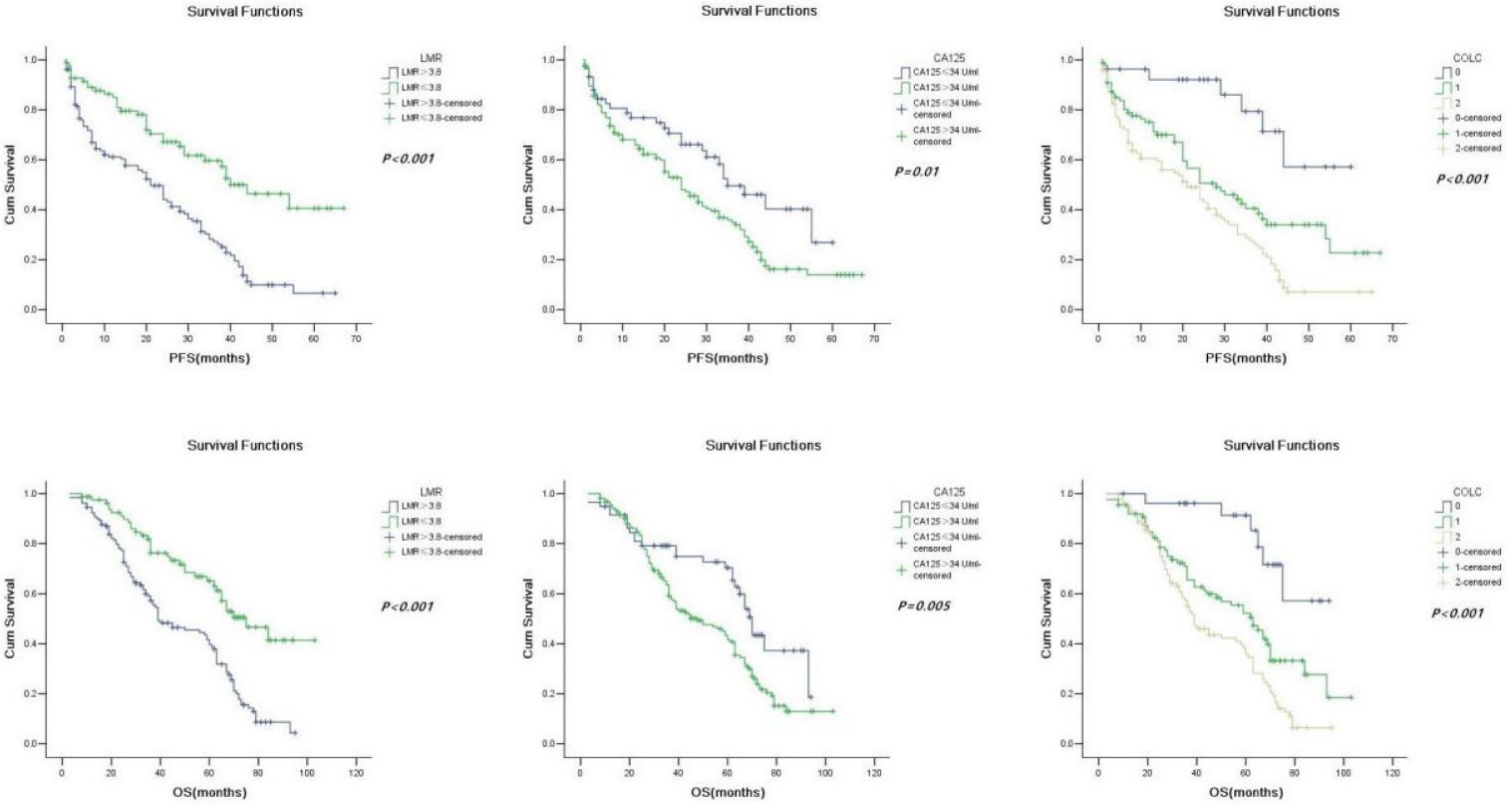
Kaplan-Meier progression-free survival curves showing the difference between the high LMR and low LMR groups (a), high CA125 and low CA125 groups (b), and COLC groups (c) (n = 0, 1, and 2) in OC patients. Kaplan-Meier overall survival curves showing the difference between the high LMR and low LMR groups (d), high CA125 and low CA125 groups (e), and COLC groups (f) (n = 0, 1, and 2) in OC patients. LMR, lymphocyte/monocyte ratio; CA125, cancer antigen 125; COLC, combination of LMR and CA125.

Patients in the low-LMR group and high-CA125 group had a significantly shorter PFS (21 months vs. 40 months, P < 0.001; 24 months vs. 35 months, P = 0.01; Fig. 2a and 2b) and OS (39 months vs. 75 months, P < 0.001; 48 months vs. 70 months, P < 0.001) than those in the high-LMR group and low-CA125 group (Fig. 2c and 2d).

Correlations of clinicopathological factors with PFS and OS were analyzed by using univariate and multivariate analyses. In univariate analyses Cox regression model, age, BMI, FIGO stage, histological subtype and grade, optimal debulking, malignant ascites, lymph node metastases, LMR, CA125 were all significantly associated with PFS (Table 2) and OS (Table 3). In the multivariate Cox regression model, FIGO stage (HR, 6.609; 95% CI: 4.013-10.880, P< 0.001), histological grade (HR = 1.943, 95% CI: 1.126-3.353, P = 0.017), optimal debulking (HR = 2.662, 95% CI: 1.804-3.927, P < 0.001), lymph node metastases (HR = 1.832, 95% CI: 1.167-2.876, P = 0.008), LMR (HR = 0.494, 95% CI: 0.329-0.742, P = 0.001), and CA125 (HR = 1.641, 95% CI: 1.057-2.550, P = 0.027) were significantly associated with PFS (Table 2); FIGO stage (HR = 7.643, 95% 95% CI: 4.797-12.175, P < 0.001), histological grade (HR = 3.672, 95% CI: 2.069-6.517, P <0.001), optimal debulking (HR = 2.662, 95% CI: 1.804-3.927, P < 0.001), lymph node metastases (HR = 2.369, 95% CI: 1.515-3.705, P <0.001), LMR (HR = 0.459, 95% CI: 0.306-0.688, P <0.001), and CA125 (HR = 1.946, 95% CI: 1.256-3.015, P = 0.003) were significantly associated with OS (Table 3).

### The Capacity of the Prognostic Value of COLC in OC

As shown above, both LMR and CA125 were prognostic markers of OC. But whether COLC had the same efficacy needed to be clarified. The definition of COLC was according to the Glasgow prognostic score (Table 4). The ROC curve of COLC in predicting mortality of OC patients was constructed (Fig. 1c). The capacities of LMR, CA125, and COLC in predicting mortality of OC patients were compared by ROC curves. The result showed that the AUC of COLC was 0.713 (95% CI: 0.641-0.786, Fig.1c)), which was higher than that of LMR (0.709, Fig.1a) and CA125 (0.583, Fig.1b). And the sensitivity and specificity of COLC were 59.3% and 75.9%.

Furthermore, univariate Cox regression and Kaplan-Meier method were used to evaluate the value of COLC in predicting OC (Fig. 2e, 2f). The result of univariate Cox regression showed that PFS (HR = 1.994, 95% CI: 1.517-2.620, P < 0.001) and OS (HR = 1.974, 95% CI: 1.502-2.595, P < 0.001) were different among the three groups (COLC = 0, 1, and 2) of OC patients.

## Discussion

The present study reported that inflammation- related factors, LMR is associated with the survival results of OC 13-15; however, consensus has not yet been reached. In our study, the cutoff value of LMR to predict the mortality of OC was close to that in the study by Zhang W 13, which indicated that LMR might be used as a reference to evaluate the outcome of OC. Besides, to further evaluate the prognostic value of LMR in OC, univariate and multivariate Cox regression analysis were performed, and the results showed that low LMR was an independent predictive factor of poor PFS and OS in OC. Furthermore, we recommended COLC to predict the survival results of OC to improve the accuracy. COLC achieved a higher AUC and specificity than LMR or CA125, indicating that COLC might have a higher specificity to predict the outcome.

On the one hand, this study provides further support for the proposition that elevated preoperative low LMR is associated with poor prognosis in OC patients. LMR has the potential to be widely used for its easier to measure, low price, and strong practicability. However, the potential mechanisms underlying the prognostic capacity of LMR have not yet been clarified. We tried to explain the mechanism. Firstly, a series of studies have proven that lymphocytes play an important role in immunologic surveillance and tumor immunoediting. They can induce tumor cell apoptosis, and inhibit tumor cell proliferation and migration 19-20. Lymphocytes enter into the tumor microenvironment and evolve into tumor-infiltrating lymphocytes 21. Therefore, OC can achieve an antitumor immune reaction 22. Secondly, monocytes are derived from chemokines and cytokines of inflammation; they could be recruited to the tumor microenvironment by tumor-derived CCL 23, and they play a role in promoting tumor progression 24. Besides, inflammation can cause monocytes to be transferred from the bone marrow to the peripheral blood 25, and induce the differentiation of monocytes into tumor-associated macrophages (TAMs) 26. TAMs express tumor necrosis factor (TNF)-α and interleukin (IL)-6, which ultimately induce the development of epithelial-mesenchymal transition (EMT) 27 to play a pro-tumor inflammatory action, promote the formation of blood vessels and lymphatics, promote the growth and metastasis of tumors, and suppress the immune response 27-28. Thirdly, high value of monocytes/TAMs can promote solid tumor progression and metastasis 29. In short, LMR represents the balance of lymphocyte and monocyte levels in OC. In other words, it can represent the balance between the anti-tumor immune reaction and the pro-tumor inflammatory reaction. Low LMR represents the decreased lymphocyte counts and the increased monocyte counts in blood and tumor stoma, results in a weakened anti-tumor immune reaction and a strong pro-tumor inflammatory reaction 30-31. Thus, a low LMR would be associated with favorable tumor progression, which results in a poor prognosis.

On the other hand, similar to numerous studies 7-9, our study proved that CA125 was associated with the prognosis of OC. The probably mechanisms are as follows: CA125 can reverse the suppressive effect of Wnt pathway inhibition on cancer cell migration 9, which results in a poor prognosis; CA125 might play a broad role in humoral immune suppression in cancers by direct binding to a subset of tumor-targeting antibodies and blockade of their immune-effector function^32^; and cancer-derived serum CA125 possesses a unique and distinctive postprandial pattern; thus, postprandial increase in serum CA125 is a surrogate biomarker for early prognostic assessment of OC 9. However, as reported by Shen Y 7, the specificity of CA125 as a prognostic factor was relatively low. Therefore, we recommended the combination of CA125 and LMR (COLC). Our results showed that the AUC and specificity of COLC were higher than that of LMR or CA125 to predict the mortality in OC; thus, implying that COLC might improve the accuracy in predicting the outcome. As a new biomarker, we infer that COLC might reflect the balance of the anti-tumor immune reaction and the pro-tumor inflammatory reaction, play a role in immune surveillance, and provide novel approaches and strategies for prognosis evaluation of OC.

To our knowledge, our study is the first attempt to evaluate the role of COLC in OC patients. Nevertheless, it showed that the AUC of LMR was higher than that of CA125. The following factors may have influenced the survival results. Firstly, the study was a retrospective, single-center research institute with a relatively small sample size, but we plan to enroll more OC patients in the validation for more definite results through multi-center, randomized research institutes. Secondly, LMR is a non-specific inflammatory marker, which is affected by patients with inflammation, immune system diseases, and combined with other tumors. However, in this study, we allowed a short time interval for blood collection to exclude any possible treatment or drug interference in the results. Another limitation of this study was the inevitable error in measurement and selection. However, we strictly followed the standards and regulations, and we verified the questionable experimental data if necessary.

## Figures and Tables

**Table 1:** Clinical characteristics of patients with ovarian cancer.

Clinical Characteristic	Measure
**Age**	50 (20-88)
**BMI (kg / m2)**	22.7 (16.5-29.9)
**Histologic subtype (n (%) )**	
Serous	146 (68.2)
Endometrioid	24 (11.2)
Transitional cell	20 (9.4)
Clear cell	12 (5.6)
Mucinous	11 (5.1)
Other	1 (0.5)
**FIGO Stage (n (%) )**	
Ⅰ	61 (28.5)
Ⅱ	38 (17.8)
Ⅲ	101 (47.2)
Ⅳ	14 (6.5)
**Histological grade (n(%) )**	
G1	28 (13.1)
G2	40 (18.7)
G3	146 (68.2)
**Optimal debulking (n(%) )**	
Yes	118 (55.1)
No	96 (44.9)
**Malignant ascites (n(%) )**	
Yes	96 (44.9)
No	118 (55.1)
**Lymph node metastases (n(%) )**	
Yes	120 (56.7)
No	94 (43.3)
**State(n(%))**	
Survival	79 (36.9)
Died	135 (63.1)
**Treatment(n(%))**	
PDS	28 (13.0)
PDS+ACT	47 (22.0)
NACT+IDS+ACT	137 (64.0)
ACT	2 (1.0)
Lymphocyte*10^9	1.3 (0.3-2.80)
Monocyte*10^9	0.4 (0.001-3.1)
**LMR**	3.64 (0.48-18.00)
**CA125 (U/ml)**	230.2 (1.4-5600)
**PFS (months)**	20 (1-67)
**OS (months)**	39.5 (3-103)

BMI, body mass index; FIGO, Federation of Gynecologists and Obstetricians; PDS, primary debulking surgery; IDS, interval debulking surgery; ACT, adjuvant chemotherapy; NACT, neo-adjuvant chemotherapy; LMR, lymphocyte/monocyte ratio; CA125, cancer antigen 125; PFS, progression-free survival; OS, overall survival

**Table 2:** Univariate and multivariate Cox proportional hazards analysis of progression-free survival.

Varies	Univariate				Multivariate		
	HR	95%CI	P		HR	95%CI	P
Age	1.015	(1.002- 1.029)	0.025		1.007	(0.992- 1.022)	0.345
BMI	1.064	(1.010- 1.120)	0.019		0.970	(0.911- 1.032)	0.337
FIGO (Ⅲ-Ⅳ vs. Ⅰ-Ⅱ)	7.987	(5.078- 12.564)	<0.001		6.609	(4.013- 10.88)	<0.001
Histological subtype (serous vs. others)	1.908	(1.298- 2.806)	0.001		1.272	(0.843- 1.920)	0.252
Histological grade (G2-G3 vs. G1)	3.891	(2.346- 6.454)	<0.001		1.943	(1.126- 3.353)	0.017
Optimal debulking (yes vs. no)	1.550	(1.097- 2.190)	0.013		1.953	(1.335- 2.856)	0.001
Malignant ascites (yes vs. no)	2.191	(1.542- 3.113)	<0.001		1.184	(0.816- 1.718)	0.374
Lymph node metastases (yes vs. no)	2.499	(1.735- 3.600)	<0.001		1.832	(1.167- 2.876)	0.008
LMR (>3.8 vs. ≤3.8 )	0.401	(0.271- 0.593)	<0.001		0.494	(0.329- 0.742)	0.001
CA125(U/ml) (≤34 vs.>34)	1.719	(1.127- 2.622)	0.012		1.641	(1.057- 2.550)	0.027

The multivariate Cox regression model demonstrated that LMR≤3.8 (HR = 0.494, 95% CI: 0.329-0.742, P = 0.001), and CA125>34 U/ml (HR = 1.641, 95% CI: 1.057-2.550, P = 0.027) were significantly associated with poor PFS. BMI, body mass index; FIGO, Federation of Gynecologists and Obstetricians; LMR, lymphocyte/monocyte ratio; CA125: cancer antigen 125.

**Table 3:** Univariate and multivariate Cox proportional hazards analysis of overall survival.

Varies	Univariate				Multivariate		
	HR	95%CI	P		HR	95%CI	P
Age	1.015	(1.002- 1.029)	0.025		1.007	(0.992- 1.022)	0.358
BMI	1.064	(1.010- 1.120)	0.019		0.953	(0.897- 1.013)	0.121
FIGO (Ⅲ-Ⅳ vs. Ⅰ-Ⅱ)	7.717	(4.963- 12.000)	<0.001		7.643	(4.797- 12.175)	<0.001
Histological subtype (serous vs. others)	1.904	(1.292- 2.805)	0.001		1.262	(0.826- 1.930)	0.282
Histological grade (G2-G3 vs. G1)	4.457	(2.660- 7.466)	<0.001		3.672	(2.069- 6.517)	<0.001
Optimal debulking (yes vs. no)	1.778	(1.255- 2.520)	0.001		2.662	(1.804- 3.927)	<0.001
Malignant ascites (yes vs. no)	2.350	(1.650- 3.347)	<0.001		1.320	(0.901- 1.933)	0.154
Lymph node metastases (yes vs. no)	2.579	(1.790- 3.715)	<0.001		2.369	(1.515 -3.705)	<0.001
LMR (>3.8 vs. ≤3.8 )	0.402	(0.272- 0.594)	<0.001		0.459	(0.306- 0.688)	<0.001
CA125(U/ml) (≤34 vs.>34)	1.802	(1.181- 2.749)	0.006		1.946	(1.256- 3.015)	0.003

The multivariate Cox regression model demonstrated that LMR≤3.8 (HR = 0.459, 95% CI: 0.306-0.688, P = <0.001), and CA125>34 U/ml (HR = 1.946, 95% CI: 1.256-3.015, P = 0.003) were significantly associated with OS. BMI, body mass index; FIGO, Federation of Gynecologists and Obstetricians; LMR, lymphocyte/monocyte ratio; CA125: cancer antigen 125.

**Table 4:** Prognosis scores of LMR, CA125 and COLC

	Score
**LMR**	
≤3.8	1
> 3.8	0
**CA125 (U/ml)**	
≤34	0
> 34	1
**COLC**	
LMR > 3.8 and CA125 < 34U/ml	0
LMR ≤ 3.8 or CA125 > 34U/ml	1
LMR ≤ 3.8 and CA125 > 34U/ml	2

LMR, lymphocyte/monocyte ratio; CA125, cancer antigen 125; COLC, combination of LMR and CA125

## References

[1] Hua J, Liu J, Hua M (2019). Diagnostic performance of biomarkers for ovarian cancer: Protocol for an overview, evidence mapping, and adjusted indirect comparisons. Medicine (Baltimore).

[2] Siegel RL, Miller KD, Jemal A (2019). Cancer statistics, 2019. CA Cancer J Clin.

[3] Torre LA, Trabert B, DeSantis CE (2018). Ovarian cancer statistics, 2018. CA Cancer J Clin.

[4] Morgan RJ, Armstrong DK, Alvarez RD (2016). Ovarian Cancer, Version 1.2016, NCCN Clinical Practice Guidelines in Oncology. J Natl Compr Canc Netw.

[5] Liu TW, Stewart JM, Macdonald TD (2013). Biologically-targeted detection of primary and micro-metastatic ovarian cancer. Theranostics.

[6] Galvan-Turner VB, Chang J, Ziogas A (2015). Observed-to-expected ratio for adherence to treatment guidelines as a quality of care indicator for ovarian cancer. Gynecol Oncol.

[7] Shen Y, Li L (2016). Serum HE4 superior to CA125 in predicting poorer surgical outcome of epithelial ovarian cancer. Tumour Biol.

[8] Zwakman N, van de Laar R, Van Gorp T (2017). Perioperative changes in serum CA125 levels: a prognostic factor for disease-specific survival in patients with ovarian cancer. J Gynecol Oncol.

[9] Yuan Q, Song J, Yang W (2017). The effect of CA125 on metastasis of ovarian cancer: old marker new function. Oncotarget.

[10] Abe S, Kawai K, Nozawa H (2018). LMR predicts outcome in patients after preoperative chemoradiotherapy for stage Ⅱ-Ⅲ rectal cancer. J Surg Res.

[11] Kumarasamy C, Sabarimurugan S, Madurantakam RM (2019). Prognostic significance of blood inflammatory biomarkers NLR, PLR, and LMR in cancer-A protocol for systematic review and meta-analysis. Medicine (Baltimore).

[12] Zhang W, Ye B, Liang W (2017). Preoperative prognostic nutritional index is a powerful predictor of prognosis in patients with stage Ⅲ ovarian cancer. Sci Rep.

[13] Zhang Y, Zhou GQ, Liu X (2016). Exploration and Validation of C-Reactive Protein/Albumin Ratio as a Novel Inflammation-Based Prognostic Marker in Nasopharyngeal Carcinoma. J Cancer.

[14] Gong J, Jiang H, Shu C (2019). Prognostic value of lymphocyte-to-monocyte ratio in ovarian cancer: a meta-analysis. J Ovarian Res.

[15] Lu C, Zhou L, Ouyang J (2019). Prognostic value of lymphocyte-to-monocyte ratio in ovarian cancer: A meta-analysis. Medicine (Baltimore).

[16] Komura N, Mabuchi S, Yokoi E (2018). Comparison of clinical utility between neutrophil count and neutrophil-lymphocyte ratio in patients with ovarian cancer: a single institutional experience and a literature review. Int J Clin Oncol.

[17] Nick AM, Coleman RL, Ramirez PT (2015). A framework for a personalized surgical approach to ovarian cancer. Nat Rev Clin Oncol.

[18] Eo WK, Chang HJ, Kwon SH (2016). The lymphocyte-monocyte ratio predicts patient Survival and Aggressiveness of Ovarian Cance. J Cancer.

[19] Merlo A, Dalla Santa S, Dolcetti R (2016). Reverse immunoediting: When immunity is edited by antigen. Immunol Lett.

[20] Gebhardt T, Palendira U, Tscharke DC (2018). Tissue-resident memory T cells in tissue homeostasis, persistent infection, and cancer surveillance. Immunol Rev.

[21] Jakubowska K, Kisielewski W, Kańczuga-Koda L (2017). Stromal and intraepithelial tumor-infiltrating lymphocytes in colorectal carcinoma. Oncol Lett.

[22] Chen ML, Yan BS, Lu WC (2014). Sorafenib relieves cell-intrinsic and cell-extrinsic inhibitions of effector T cells in tumor microenvironment to augment antitumor immunity. Int J Cancer.

[23] Schierer S, Ostalecki C, Zinser E (2018). Extracellular vesicles from mature dendritic cells (DC) differentiate monocytes into immature DC. Life Sci Alliance.

[24] Hamm A, Prenen H, Van Delm W (2016). Tumour-educated circulating monocytes are powerful candidate biomarkers for diagnosis and disease follow-up of colorectal cancer. Gut.

[25] Shi C, Pamer EG (2011). Monocyte recruitment during infection and inflammation. Nat Rev Immunol.

[26] Wang F, Li B, Wei Y (2018). Tumor-derived exosomes induce PD1+macrophage population in human gastric cancer that promotes disease progression. Oncogenesis.

[27] Xu S, Liu Z, Lv M (2019). Intestinal dysbiosis promotes epithelial-mesenchymal transition by activating tumor-associated macrophages in ovarian cancer.

[28] Jeong H, Hwang I, Kang SH (2019). Tumor-Associated Macrophages as Potential Prognostic Biomarkers of Invasive Breast Cancer. J Breast Cancer.

[29] Franklin RA, Liao W, Sarkar A (2014). The cellular and molecular origin of tumor-associated macrophages. Science.

[30] Patel M, McSorley ST, Park JH (2018). The relationship between right-sided tumour location, tumour microenvironment, systemic inflammation, adjuvant therapy and survival in patients undergoing surgery for colon and rectal cancer. Br J Cancer.

[31] Zhang Y, Wang L, Liu Y (2014). Preoperative neutrophil-lymphocyte ratio before platelet-lymphocyte ratio predicts clinical outcome in patients with cervical cancer treated with initial radical surgery. Int J Gynecol Cancer.

[32] Nicolaides NC, Schweizer C, Somers EB, et L (2018). CA125 suppresses amatuximab immune-effector function and elevated serum levels are associated with reduced clinical response in first line mesothelioma patients. Cancer Biol Ther.

